# Hepatitis B and C viruses and risk of non-Hodgkin lymphoma: a case-control study in Italy

**DOI:** 10.1186/s13027-016-0073-x

**Published:** 2016-06-23

**Authors:** Martina Taborelli, Jerry Polesel, Maurizio Montella, Massimo Libra, Rosamaria Tedeschi, Monica Battiston, Michele Spina, Francesco Di Raimondo, Antonio Pinto, Anna Crispo, Maria Grimaldi, Silvia Franceschi, Luigino Dal Maso, Diego Serraino

**Affiliations:** Unit of Cancer Epidemiology, CRO Aviano National Cancer Institute, via Franco Gallini 2, 33081 Aviano, Italy; Unit of Epidemiology, National Cancer Institute “G. Pascale Foundation”, Cappella dei Cangiani, 80131 Naples, Italy; Department of Biomedical Sciences, University of Catania, via Androne 83, 95124 Catania, Italy; Unit of Microbiology, Immunology and Virology, CRO Aviano National Cancer Institute, via Franco Gallini 2, 33081 Aviano, Italy; Unit of Stem Cells Collection and Processing Unit for Cells Therapy, CRO Aviano National Cancer Institute, via Franco Gallini 2, 33081 Aviano, Italy; Unit of Medical Oncology A, CRO Aviano National Cancer Institute, via Franco Gallini 2, 33081 Aviano, Italy; Division of Hematology, Azienda Policlinico-OVE, University of Catania, via Citelli 6, 95124 Catania, Italy; UOSC di Ematologia Oncologica, National Cancer Institute “G. Pascale Foundation”, via M. Semmola, 80131 Naples, Italy; International Agency for Research on Cancer, 150 Cours Albert Thomas, 69008 Lyon, France

**Keywords:** Non-Hodgkin lymphoma, Hepatitis C virus, Hepatitis B virus, Case-control study

## Abstract

**Background:**

Hepatitis C virus (HCV) has been consistently associated to non-Hodgkin lymphoma (NHL); conversely, few studies have evaluated a comprehensive serological panel of hepatitis B virus (HBV) in NHL etiology.

**Methods:**

We conducted a case-control study in Italy in 1999–2014, enrolling 571 incident, histologically confirmed NHLs and 1004 cancer-free matched controls. Study subjects provided serum for HCV and HBV testing and for HCV RNA. Odds ratios (ORs) and corresponding 95 % confidence intervals (CIs) were estimated by logistic regression, adjusting for potential confounders.

**Results:**

Circulating HCV RNA was detected in 63 (11.1 %) NHL cases and 35 (3.5 %) controls (OR = 3.51, 95 % CI: 2.25–5.47). Chronic HBV infection (i.e., positive to HBV surface antigen - HBsAg^+^) was found in 3.7 % of cases and 1.7 % of controls (OR = 1.95, 95 % CI: 1.00–3.81); a significantly elevated OR was observed for B-cell NHL (2.11, 95 % CI: 1.07–4.15). People with serological evidence of past HCV or HBV infection, vaccination against HBV, or detectable antibodies against HBV core antigen (anti-HBc^+^) alone were not at increased NHL risk.

**Conclusions:**

Our results support a role of chronic HCV infection in NHL in Italy and suggest an involvement of HBV infection. Associations were clearest for B-cell NHL and diffuse large B-cell lymphoma. Prevention and treatment of HCV and HBV infection may diminish NHL incidence, notably in areas with high prevalence of hepatitis viruses infection.

## Background

Non-Hodgkin lymphomas (NHLs) are the most frequent haematological malignancies, representing 3 % of all new cancer cases worldwide [1]. In Europe, NHLs represented the 11th most common neoplasm, with around 93,500 new cases diagnosed in 2012. Among European countries, Italy shows one of the highest age-standardized incidence rates in both men and women [2].

Although the etiology of most NHL cases is still unknown, human immunodeficiency virus (HIV) and other infectious agents such as Epstein-Barr virus, human herpesvirus 8, human T-lymphotropic virus type I, and *Helicobacter pylori* are the best documented causes of specific types of NHL [3, 4].

Despite the etiologic heterogeneity among NHL subtypes [5], a consistent association between hepatitis C virus (HCV) infection and NHL has been well established over the last two decades [3, 6, 7], suggesting that, globally, approximately 8 % of NHL may be attributable to HCV [8]. Few studies have evaluated the possible role of hepatitis B virus (HBV) infection as a risk factor for NHL and showed positive association in some but not all instances [9]. Furthermore, the majority of these investigations lacked information on the complete panel of antigen and antibody markers of HBV infection [10–12].

To further elucidate the relationship between HBV and HCV infections and the risk of NHLs, we expanded a case-control study, which had started in 1999, in different areas of Italy [13]. Special care was put into exploring a comprehensive HBV serologic panel to allow a well-defined assessment of its role in the risk of developing NHL.

## Methods

The data in the present study were derived from two consecutive case-control studies on lymphomas, conducted with similar study protocols in the periods 1999–2002 [13] and 2003–2014.

### First study, 1999–2002

Between 1999 and 2002, we conducted a multi-cancer case-control study on the association between HBV/HCV infections and lymphomas and hepatocellular carcinoma (HCC) in the province of Pordenone, northeastern Italy, and the town of Naples, southern Italy. The study design and findings are described elsewhere [13, 14]. Briefly, the study included 231 cases with incident, histologically confirmed NHL aged 18–84 years (median age: 59 years). Controls were 547 inpatients aged 18–84 years (median age: 62 years) admitted for a wide spectrum of acute conditions to the same hospitals as cases. They were frequency-matched according to center (Pordenone, Naples), gender, and age (in 5-year age groups) based on the distribution of overall study cases, which also included Hodgkin lymphomas (HL) and HCCs. Consequently, as already reported [13], controls were younger and more likely to be men slightly than NHL cases. Specifically excluded from the control group were patients admitted for malignant diseases, conditions related to alcohol and tobacco consumption or hepatitis viruses as well as any chronic diseases that might have changed lifestyle habits, hematologic, allergic, and autoimmune diseases. However, comorbidity for the above listed diseases was not an exclusion criterion.

### Second study, 2003–2014

Between 2003 and 2014, we extended the previous study, focusing only on lymphomas, and maintaining the same study design, inclusion and exclusion criteria, and questionnaire. Cases were 353 patients aged 18–84 years (median age: 56 years) with incident, histologically confirmed NHL. They were admitted to two National Cancer Institutes located in Aviano (“Centro di Riferimento Oncologico”) and in Naples (“Fondazione G. Pascale”), and to the general hospitals located in Catania. The control group included 537 patients aged 18–83 years (median age: 50 years), admitted for a wide spectrum of acute conditions to the same hospitals as lymphomas cases. Cases and controls were frequency-matched by center (Pordenone, Naples, and Catania), gender, and age (in 5-year age groups) based on the distribution of both HL and NHL cases.

In order to guarantee a sufficient statistical power, particularly in respect to NHL subtypes and different combinations of viral markers, the two studies were combined. Overall, a total of 584 NHL cases and 1084 controls participated in the two studies. Thirteen cases were interviewed but could not give blood samples, leaving 571 NHL cases (median age: 56 years) with available questionnaires and blood samples. Histological diagnoses were centrally revised, and cases were classified according to the International Classification of Diseases for Oncology (third edition) [15]. Blood samples were available for 1004 controls (median age: 57 years) of whom, 20.4 % were admitted to the hospital for trauma, 39.4 % for nontraumatic orthopoedic diseases, 20.9 % for acute surgical conditions, 9.2 % for eye diseases, and 10.1 % for a variety of other illnesses. All NHL cases were tested for HIV as part of their routine management, and they were all HIV-negative. To the best of our knowledge, no control subjects had a history of HIV infection or AIDS.

All study participants signed an informed consent, according to the recommendations of the Board of Ethics (CRO Aviano National Cancer Institute Board of Ethics; National Cancer Institute "G. Pascale" Board of Ethics; University of Catania Board of Ethics) of each study center, which had approved the study.

### Questionnaire

Trained interviewers administered a structured questionnaire to cases and controls during their hospital stay. It included information on socio-demographic indicators, tobacco smoking, alcohol drinking, dietary habits, behaviors, major exposures that entailed increased risk of HCV/HBV transmission and history of diseases affecting the immune system.

### HCV and HBV testing

Each case and each control provided a 15 ml blood sample (7.5 ml in vacutainer tubes with anticoagulant and 7.5 ml in vacutainer tubes without anticoagulant) the day of interview (generally the first day of hospital stay). Blood samples were centrifuged at 1500 g for 10 min, extracted and distributed into different cryotubes of serum, buffy coat, and red blood cells, and then stored at −80 °C until shipment to the laboratory of Microbiology, Immunology and Virology, National Cancer Institute of Aviano, where they were tested in a blind fashion.

### HCV

Sera were screened for antibodies against HCV (anti-HCV) using a third-generation chemiluminescent microparticle immunoassay (CMIA ARCHITECT HCV Ag Assay, Abbott Diagnostic Division, Wiesbaden, Germany). The assay detects antibodies to both structural (core region) and non-structural proteins of the HCV genome with sensitivity and specificity estimated to be above 99 %. Anti-HCV positive samples were tested for serum HCV RNA using the Abbott HCV RealTime (ART, Abbott Diagnostic Division, Wiesbaden, Germany) with a limit of detection of 12 IU/mL. Samples were considered HCV-positive if HCV antibodies and HCV RNA were detected.

### HBV

Testing for antibodies to HBV surface antigen (anti-HBs) and antibodies to HBV core antigen (anti-HBc) was performed using CMIA (ARCHITECT anti-HBs and ARCHITECT anti-HBc, Abbott Diagnostic Division, Wiesbaden, Germany). Samples giving borderline test results were retested. HBV surface antigen (HBsAg) was tested using CMIA (ARCHITECT HBsAg Abbott Diagnostic Division, Wiesbaden, Germany) and confirmed by neutralization test (AxSYM, Abbott Diagnostic Division, Wiesbaden, Germany). Samples were deemed HBsAg^+^ when positive at the neutralization test. Study subjects without available information on all three HBV markers (one case and seven controls) were excluded from analyses on HBV markers.

### Statistical methods

Adjusted odds ratios (ORs) and corresponding 95 % confidence intervals (95 % CIs) were estimated by unconditional multiple logistic regression, including terms for matching variables –i.e., gender, age (in quinquennia plus a term for age as a continuous variable), study center– and potential socio-demographic determinants (i.e., years of education and place of birth) [16].

## Results

Table 1 shows the distribution of NHL cases and controls from both consecutive studies by study center, calendar year, gender, age, place of birth, and education. People born in southern Italy were at elevated NHL risk (OR = 2.35, 95 % CI: 1.60–3.47), as compared to those born in northern or central Italy. Education level was unrelated to NHL risk.

**Table 1:** Distribution of 571 NHL and 1004 controls with ORs and 95 % CIs for selected characteristics. Italy, 1999–2014

	NHL	Controls	
	N (%)	N (%)	OR (95 % CI)^a^
Study center			
Aviano	272 (47.6)	527 (52.5)	
Napoli	215 (37.7)	359 (35.8)	
Catania	84 (14.7)	118 (11.7)	
Year of interview			
1999–2002	228 (39.9)	504 (50.2)	
2003–2014	343 (60.1)	500 (49.8)	
Gender			
Male	318 (55.7)	622 (62.0)	
Female	253 (44.3)	382 (38.0)	
Age (years)			
<45	137 (24.0)	305 (30.4)	
45–64	260 (45.5)	366 (36.4)	
≥65	174 (30.5)	333 (33.2)	
Place of birth^c^			
North-Center	221 (38.8)	487 (48.6)	1^b^
South	349 (61.2)	515 (51.4)	2.35 (1.60–3.47)
Education (years)^c^			
<7	195 (34.2)	368 (36.6)	1^b^
7–11	186 (32.6)	300 (29.9)	1.27 (0.96–1.70)
≥12	189 (33.2)	336 (33.5)	1.20 (0.90–1.60)

*NHL* non-Hodgkin lymphoma, *OR* odds ratio, *CI* confidence interval

^a^Estimated using an unconditional logistic regression model adjusted for gender, age, study center, years of education, and place of birth; ^b^Reference category; ^c^The sum does not add up to the total because of some missing values

The association between HCV and HBV serologic markers and NHL risk is shown in Table 2. Altogether, 79 NHL cases (13.8 %) and 64 controls (6.4 %) were anti-HCV^+^ (OR = 2.39, 95 % CI: 1.66–3.43). In the confirmatory analysis with HCV RNA, 63 NHL patients (11.1 %) and 35 controls (3.5 %) were found positive, leading to a 3.5-fold higher risk of NHL in people positive for HCV RNA^+^ (95 % CI: 2.25–5.47) as compared to anti-HCV^−^. Conversely, no excess of NHL risk emerged among anti-HCV^+^/HCV RNA^−^ people (OR = 0.98, 95 % CI: 0.50–1.92).

**Table 2:** ORs and 95 % CIs for NHL by markers of HCV and HBV. Italy, 1999–2014

	NHL	Controls	
	N (%)	N (%)	OR (95 % CI)^a^
HCV	571	1004	
Anti-HCV^-^	492 (86.2)	940 (93.6)	1^b^
Anti-HCV^+^	79 (13.8)	64 (6.4)	2.39 (1.66–3.43)
*HCV RNA* ^*-*c^	*14 (2.5)*	*27 (2.7)*	*0.98 (0.50–1.92)*
*HCV RNA* ^*+*^	*63 (11.1)*	*35 (3.5)*	*3.51 (2.25–5.47)*
HBV^d^	570	997	
HBsAg^-^/anti-HBs^-^/anti-HBc^-^	359 (63.0)	609 (61.1)	1^b^
HBsAg^-^/anti-HBs^+^/anti-HBc^+^	90 (15.8)	179 (18.0)	0.78 (0.58–1.06)
HBsAg^-^/anti-HBs^+^/anti-HBc^-^	53 (9.3)	122 (12.2)	0.87 (0.59–1.29)
HBsAg^-^/anti-HBs^-^/anti-HBc^+^	47 (8.2)	70 (7.0)	1.10 (0.73–1.65)
HBsAg^+^	21 (3.7)	17 (1.7)	1.95 (1.00–3.81)

*NHL* non-Hodgkin lymphoma, *OR* odds ratio, *CI* confidence interval

^a^Estimated using unconditional logistic regression models adjusted for gender, age, study center, years of education, and place of birth; ^b^Reference category; ^c^Information on HCV RNA was missing for 2 cases and 2 controls; ^d^1 case and 7 controls without complete information on all HBV markers were excluded

Approximately 60 % of both NHL cases and controls tested negative for any HBV serologic markers (Table 2). The prevalence of HBsAg seropositivity was 3.7 % among NHL cases and 1.7 % among controls, leading to an OR of 1.95 (95 % CI: 1.00–3.81) for HBsAg^+^ individuals compared to people susceptible to HBV infection. Immunity due to natural infection (i.e., HBsAg^−^/anti-HBs^+^/anti-HBc^+^), immunity due to vaccination (HBsAg^-^/anti-HBs^+^/anti-HBc^-^), and the positivity for anti-HBc alone (HBsAg^−^/anti-HBs^−^/anti-HBc^+^) were not significantly associated with NHL risk. Chronic co-infection of HBV and HCV was reported in only one NHL case.

Subgroup analyses of the association between NHL and the presence of HCV RNA or HBsAg were performed for the main histological subtypes (Table 3). The prevalence of HCV RNA positivity was 11.7 % among B-cell NHL subtypes, whereas only two out of 37 (5.4 %) T-cell NHL cases were found to be positive for HCV RNA. HCV RNA^+^ people, as compared to anti-HCV^−^, showed an elevated risk of diffuse large B-cell lymphoma (DLBCL; OR = 4.08, 95 % CI: 2.46–6.76), marginal zone lymphoma (MZL; OR = 8.62, 95 % CI: 3.43–21.69), lymphoplasmacytic lymphoma (LPL, OR = 8.43, 95 % CI: 2.26–31.52), and small lymphocytic lymphoma/chronic lymphocytic leukemia (SLL/CLL; OR = 7.96, 95 % CI: 2.95–21.48). Regarding HBV, the proportion of HBsAg-positive cases was 3.9 % among B-cell NHL and 0.0 % among T-cell NHL. HBsAg positivity was associated with a significantly increased risk of B-cell NHL subtypes overall (OR = 2.11, 95 % CI: 1.07–4.15) and of DLBCL in particular (OR = 2.69, 95 % CI: 1.30–5.59). Neither HCV nor HBV infections were significantly associated with the risk of follicular NHL (FL).

**Table 3:** ORs and 95 % CIs for selected NHL histological types by HCV RNA/HBsAg positivity. Italy, 1999–2014

	All^c^	HCV-RNA^+^		All^d^	HBsAg^+^	
	N	N (%)	OR (95 % CI)^a,e^	N	N (%)	OR (95 % CI)^a,f^
Controls	1002	35 (3.5)	1^b^	997	17 (1.7)	1^b^
NHL cases	569	63 (11.1)		570	21 (3.7)	
B-cell lymphomas	512	60 (11.7)	3.71 (2.37–5.81)	513	20 (3.9)	2.11 (1.07–4.15)
*DLBCL*	*289*	*37 (12.8)*	*4.08 (2.46–6.76)*	*290*	*15 (5.2)*	*2.69 (1.30–5.59)*
*Burkitt*	*14*	*2 (14.3)*	*4.36 (0.82–23.26)*	*14*	*0 (0.0)*	
*Follicular*	*103*	*1 (1.0)*	*0.38 (0.05–2.85)*	*103*	*4 (3.9)*	*1.96 (0.61–6.29)*
*Mantle cell*	*14*	*0 (0.0)*		*14*	*0 (0.0)*	
*Marginal zone*	*33*	*9 (27.3)*	*8.62 (3.43–21.69)*	*33*	*0 (0.0)*	
*Lymphoplasmacytic*	*14*	*4 (28.6)*	*8.43 (2.26–31.52)*	*14*	*0 (0.0)*	
*Other B-cell lymphomas*	*10*	*0 (0.0)*		*10*	*0 (0.0)*	
*SLL/CLL*	*35*	*7 (20.0)*	*7. 96 (2.95–21.48)*	*35*	*1 (2.9)*	*1.18 (0.14–10.05)*
T-cell lymphomas	37	2 (5.4)	1.68 (0.37–7.69)	37	0 (0.0)	
Other	2	0 (0.0)		2	0 (0.0)	
NOS	18	1 (5.6)	1.73 (0.20–15.01)	18	1 (5.5)	2.58 (0.28–23.84)

*NHL* non-Hodgkin lymphoma, *OR* odds ratio, *CI* confidence interval, *DLBCL* diffuse large B-cell lymphoma, *SLL* small lymphocytic lymphoma, *CLL* chronic lymphocytic leukemia, *NOS* not otherwise specified; ^a^Estimated using unconditional logistic regression models adjusted for gender, age, study center, years of education, and place of birth; ^b^Reference category. ^c^Cases and controls with positive anti-HCV test and without available information on HCV-RNA were excluded; ^d^Cases and controls without available information on HBV markers were excluded; ^e^Reference category was anti-HCV^-^; ^f^Reference category was HBsAg^-^/anti-HBs^-^/anti-HBc^-^

## Discussion

The findings from this large case-control study confirmed the well-established association between HCV infection and NHL, highlighting the potential favorable role of prevention and treatment of HCV infection. The study also provided further evidence on the role of HBV infection in NHL etiology, reporting a 2-fold significantly elevated risk of B-cell NHL in HBsAg^+^ people. No increased risk emerged among people immunized because of past infection or vaccination, or among those who tested positive for anti-HBc alone.

Although chronic HCV infection has been associated to NHL since the early 1990s [17, 18], the International Agency for Research on Cancer definitely linked it to NHL etiology only in 2009 [3]. These findings were further supported by a recent pooled analysis of case-control studies from the InterLymph Consortium [5], which reported an 1.8-fold increase in NHL risk for anti-HCV^+^ people.

Although anti-HCV-positivity was associated to NHL risk, we found that only individuals with circulating HCV RNA were actually at increased risk for NHL, indicating that only persistent chronic HCV infection is associated to lymphomagenesis. This finding is in agreement with a previous large multicenter case-control study from EPILYMPH [19], which found a statistically significant association between NHL and detectable HCV RNA (OR = 1.82, 95 % CI: 1.13–2.91).

The association with HCV-positivity was found for a broad range of B-cell NHL subtypes, with a high prevalence of chronically infected NHL patients (13–29 %) in our study. Our results are in agreement with several studies that found an association of HCV with DLBCL, MZL, LPL, and SLL/CLL [7, 19–24]. At variance with a previous study [25], no significant association emerged between HCV infection and Burkitt lymphoma. Prior studies have produced mixed results for T-cell NHL [6], but no significant relationship with HCV infection emerged from our study.

The association between HCV infection and B-cell NHL found in this study is consistent with the evidence that proliferation of specific B-cell clones, caused by chronic antigenic stimulation induced by HCV, plays a role in the pathogenesis of B-cell lymphomas [26]. In fact, continuous stimulation of the immune system by HCV may lead to molecular alterations in the lymphocytes, immune dysregulation, and eventually B-cell [27].

HBV infection has been studied less frequently than HCV infection in relation to NHLs, and most of the evidence is based on case–control studies [3, 9]. Furthermore, although a comprehensive testing of HBV antigens and antibodies is required to assess the HBV status, only few studies considered the complete HBV serological panel. In our study, a two-fold increased NHL risk was found among HBsAg^+^ patients. The association was statistically significant for B-cell NHL. A previous Italian study on B-cell lymphomas [28] reported complete information on HBV serology, showing a pattern of association between HBV status and NHL very similar to our present findings. Conversely, non-significant associations were reported by the Epilymph case-control study [29] for both chronic and past HBV infections.

The majority of the studies have considered the HBV markers separately, most of them resting on HBsAg. Findings on HBsAg^+^ have been associated to a 1.5-to-4-fold increase in NHL risk [10, 12, 13, 30, 31]. Seropositivity to anti-HBc alone has been investigated quite rarely as a marker of past HBV infection, reporting a moderate or non-significant association with NHLs [12].

Although our results should be interpreted with caution as they are based on a relatively small number of HBV carriers, we found that HBsAg^+^ patients had an elevated risk for B-cell NHL and, particularly, DLBCL. In agreement with our results, a multicenter case-control study [28] found a higher prevalence of HBsAg^+^ among patients with DLBCL or FL than among controls. In South Korea, a large cohort study [30] showed that HBsAg-positivity was associated with an increased risk of DLBCL, but not with FL.

The association of NHL, and predominantly of B-cell lymphoid malignancies, with active chronic HBV infection, though not with past exposure, suggests that HBV might promote B-cell lymphomagenesis in a way similar to that proposed for HCV (i.e., chronic antigen stimulation of B-cells), possibly enhanced by engagement of B-cell surface receptors and co-stimulatory molecules [6].

The presence of occult HBV infections – i.e., the presence of circulating HBV DNA in HBsAg^−^/anti-HBs^−^ patients – is a relevant concern in etiological studies, since it may lead to biased results. Occult HBV infection was, however, rare in our study population. In a companion study on HCC, all HBsAg^−^/anti-HBs^−^ cases and controls were screened for HBV DNA [14]. HBV DNA was detected in one HCC case out of 85 HBsAg^−^/anti-HBs^−^/anti-HBc^−^ study subjects (1.2 %) and in one HCC case out of 36 HBsAg^−^/anti-HBs^−^/anti-HBc^+^ subjects (2.8 %). Both cases were co-infected with HCV.

Interpretation of study findings should take into account some limitations. First, hospital-based case-control studies are prone to selection bias in the inclusion of hospital controls. However, the prevalence of HBV and HCV positivity in the control group was consistent with the findings of previous surveys of hepatitis virus prevalence in Italy [32]. Furthermore, individuals admitted to hospital for diseases related to alcohol and tobacco consumption, or hepatitis viruses and autoimmune diseases, were excluded. Second, despite the relatively large sample size, the study has still limited power to detect association for specific NHL subtypes. Third, people with anti-HCV^−^ were not tested for HCV RNA. Although an underestimation of HCV prevalence cannot be completely ruled out, no excess of NHL risk emerged among anti-HCV^+^/HCV RNA^−^ people. Accurate testing for serological detection of HCV/HBV in a centralized laboratory and the revision of NHL diagnosis represent important strengths of our study.

## Conclusions

Our case-control study confirmed the association between HCV infection and risk of NHL, including the major NHL subtypes. Furthermore, our results lent additional support to the possibility that chronic HBV infection may also have a role in the development of B-cell NHL. Prevention and treatment of HCV and HBV infection is increasingly possible and may diminish NHL incidence, notably in areas with high prevalence of infection with hepatitis viruses.

## Abbreviations

anti-HBc: antibodies to HBV core antigen
anti-HBs: antibodies to HBV surface antigen
anti-HCV: antibodies against HCV
CI: confidence interval
CLL: chronic lymphocytic leukemia
DLBCL: diffuse large B-cell lymphoma
FL: follicular lymphoma
HBsAg: HBV surface antigen
HBV: hepatitis B virus
HCC: hepatocellular carcinoma
HCV: hepatitis C virus
HIV: human immunodeficiency virus
HL: Hodgkin lymphoma
LPL: lymphoplasmacytic lymphoma
MZL: marginal zone lymphoma
NHL: non-Hodgkin lymphoma
OR: odds ratio
SLL: small lymphocytic lymphoma
